# Perioperative Arrhythmias: Pathophysiology, Risk Stratification, Management, and Emerging Technologies—A Narrative Review Toward Personalised Care

**DOI:** 10.3390/jpm16070367

**Published:** 2026-07-04

**Authors:** Daniele Salvatore Paternò, Luigi La Via, Marco Lo Presti, Gilberto Duarte-Medrano, Natalia Nuño-Lámbarri, Emilia Concetta Lo Giudice, Giordana Russo, Mattia Pratini, Paolo Tummino, Giuseppe Scibilia, Marco Barbanti, Massimiliano Sorbello

**Affiliations:** 1 Department of Anesthesia and Intensive Care, “Giovanni Paolo II” Hospital, 97100 Ragusa, Italy; massimiliano.sorbello@unikore.it; 2 Department of General Surgery and Medical-Surgical Specialties, University of Catania, 95123 Catania, Italy; luigi.lavia@unict.it; 3 Department of Cardiology, “Umberto I” Hospital, 94100 Enna, Italy; marco.lopresti92@gmail.com (M.L.P.); marco.barbanti@unikore.it (M.B.); 4 Department of Anesthesia, Medica Sur Hospital, Mexico City 11510, Mexico; gduartem@medicasur.org.mx; 5 Translational Research Unit, Medica Sur Clinic & Foundation, Mexico City 11510, Mexico; nnunol@medicasur.org.mx; 6 Department of Surgery, The National Autonomous University of Mexico (UNAM), Mexico City 11510, Mexico; 7 School of Anesthesia and Intensive Care, “Kore” University, 94100 Enna, Italy; emilia.logiudice@unikore.it (E.C.L.G.); giordana.russo001@unikorestudent.it (G.R.); mattia.pratini@unikorestudent.it (M.P.); paolo.tummino@unikorestudent.it (P.T.); 8 Department of Obstetric and Gynecological Surgery, “Giovanni Paolo II” Hospital, 97100 Ragusa, Italy; giuseppe.scibilia@unikore.it; 9 School of Obstetric and Gynecological Surgery, “Kore” University, 94100 Enna, Italy; 10 School of Cardiology, “Kore” University, 94100 Enna, Italy

**Keywords:** perioperative arrhythmias, postoperative atrial fibrillation, risk stratification, personalised medicine, artificial intelligence, arrhythmia prophylaxis, anticoagulation

## Abstract

Cardiac arrhythmias complicate 20–50% of surgical procedures and contribute substantially to perioperative morbidity, mortality, and healthcare costs, with postoperative atrial fibrillation (POAF) being the most frequent form. Their genesis reflects the convergence of surgical stress, anaesthetic agents, autonomic imbalance, systemic inflammation, and electrolyte disturbances, explaining the limited efficacy of single-mechanism interventions. This narrative review synthesises contemporary evidence on pathophysiology, risk stratification, prevention, acute management, and emerging technologies, emphasising individualised, patient-tailored approaches. MEDLINE, Embase, and Cochrane CENTRAL were searched (January 2010–January 2026), prioritising randomised trials, meta-analyses, and guidelines. Contemporary risk stratification integrates clinical scores, biomarkers, and electrocardiographic parameters; machine-learning models show moderate discrimination (pooled AUC 0.84) and may enable more personalised prediction pending external validation. Evidence-based prophylaxis—beta-blockade, magnesium, selective amiodarone, and emerging anti-inflammatory strategies such as colchicine—reduces POAF in high-risk populations, while acute management is guided by haemodynamic status and individual risk. Anticoagulation follows CHA_2_DS_2_-VASc stratification, although optimal timing and duration remain undefined. Wearable monitoring, AI-based detection, and atrial-selective agents show clinical promise. Systematic, personalised integration of risk assessment, prophylaxis, monitoring, and management offers the clearest path to reducing arrhythmia-associated morbidity.

## 1. Introduction

Cardiac arrhythmias are among the most frequent perioperative complications, affecting 20–50% of patients undergoing major surgery [1,2]. Postoperative atrial fibrillation (POAF) is the most prevalent form and, in patients with atrial fibrillation, is independently associated with increased risks of stroke, heart failure, myocardial infarction, and all-cause mortality [3]; observational data suggest that POAF confers an analogous adverse long-term profile rather than representing a purely transient electrical phenomenon, potentially unmasking underlying cardiovascular vulnerability. The economic burden is substantial—POAF prolongs hospitalisation and markedly increases healthcare costs [4]. Contemporary surgical practice increasingly confronts ageing patients with multiple comorbidities, heightening the need for accurate, individualised prediction, prevention, and management.

Perioperative arrhythmogenesis is multifactorial. The surgical stress response drives catecholamine and cortisol surges that shorten atrial refractoriness and favour reentry; volatile and local anaesthetic agents modulate cardiac ion channels; systemic inflammation and electrolyte disturbances promote atrial remodelling and electrical heterogeneity; and haemodynamic perturbations precipitate ischaemia-related arrhythmias [2,5,6,7,8,9,10]. These mechanisms interact synergistically in susceptible individuals and explain why single-mechanism interventions typically achieve only modest efficacy.

Management has shifted from reactive treatment toward proactive, risk-based prevention. Machine-learning models augment conventional scores in arrhythmia prediction, with a systematic review and meta-analysis reporting moderate pooled discrimination (AUC 0.84) for postoperative atrial fibrillation after coronary artery bypass grafting [11], while real-time ECG analysis can flag subtle patterns preceding clinical onset [12,13]. Wearable devices enable post-discharge surveillance [14], atrial-selective agents offer improved safety profiles [15], and the 2023 ACC/AHA/ACCP/HRS guideline [16] emphasises individualised strategies and recognises POAF as a long-term cardiovascular risk marker.

Despite this progress, evidence on perioperative arrhythmias remains fragmented across specialties, and the integration of AI-based, biomarker-informed, and patient-specific approaches into a coherent perioperative framework has not been systematically synthesised. This narrative review addresses that gap by organising current evidence around an individualised, patient-tailored model of care. We examine the multifactorial pathophysiology; contemporary risk stratification—including clinical scores, biomarkers, electrocardiographic parameters, AI, and underappreciated modifiers such as sex and frailty; the electrophysiological effects of anaesthetic agents; evidence-based prophylaxis, intraoperative monitoring, and acute management; postoperative care with particular attention to anticoagulation; special populations (patients with cardiac implantable electronic devices and inherited arrhythmia syndromes); and emerging technologies. Targeting anaesthesiologists, surgeons, cardiologists, electrophysiologists, and intensivists, the review prioritises evidence from randomised controlled trials (RCTs), meta-analyses, and clinical guidelines while acknowledging key knowledge gaps and research priorities.

## 2. Methods

### 2.1. Study Design and Literature Search

This is a narrative review based on a structured, systematic literature search. We searched MEDLINE (PubMed), Embase, and Cochrane CENTRAL from January 2010 to January 2026 (last searched January 2026), using strategies combining MeSH terms and free-text keywords. Primary terms included “Arrhythmia,” “Atrial fibrillation,” “Perioperative,” and “Anaesthesia.” Secondary terms encompassed “Risk stratification,” “Prophylaxis,” “Beta-blockers,” “Amiodarone,” “Magnesium,” “Cardioversion,” “Anticoagulation,” “Artificial intelligence,” “Machine learning,” “Wearable devices,” and “Remote monitoring.” Supplementary strategies included hand-searching reference lists, reviewing clinical practice guidelines from the ACC/AHA, ESC, and ESAIC, consulting content experts, and conducting forward citation searches. Seminal references predating the search window were retained when foundational to the topic and were identified through reference-list screening.

### 2.2. Selection Criteria

Studies were included if they comprised RCTs, systematic reviews, meta-analyses, or clinical practice guidelines; large observational cohort studies (n ≥ 500); or relevant case–control studies. Eligible populations were adult surgical patients (aged ≥18 years) undergoing cardiac or non-cardiac surgery. Covered topics included anaesthetic effects on cardiac electrophysiology, arrhythmia prophylaxis, intraoperative monitoring, acute management, anticoagulation, and emerging technologies. English-language publications were prioritised, with non-English sources considered where they represented unique high-quality evidence. Studies were excluded if they involved exclusively paediatric populations; comprised case reports or series of fewer than 10 patients (except for rare conditions); were non-peer-reviewed (except expert consensus statements); or addressed non-surgical critical-care arrhythmias or arrhythmias induced by exercise or medications outside perioperative contexts.

### 2.3. Data Synthesis

A narrative synthesis approach was employed. Evidence was prioritised according to study design (RCTs and meta-analyses ranked above cohort and retrospective studies), sample size, methodological rigour, clinical relevance to contemporary practice, and recency—particularly for rapidly evolving domains such as artificial intelligence. Extracted data included study design, population characteristics, interventions, outcomes with effect sizes, key findings, and methodological limitations. Synthesis was organised thematically by manuscript section, integrating guideline recommendations, acknowledging areas of controversy, and identifying knowledge gaps.

### 2.4. Limitations

Several limitations merit acknowledgement. The narrative approach prioritises breadth and clinical applicability over the exhaustive methodology of a formal systematic review or meta-analysis; accordingly, no PRISMA flow diagram or quantitative pooling was undertaken. To mitigate the inherent subjectivity of narrative synthesis, the search strategy, inclusion and exclusion criteria, and thematic structure of the review were defined a priori through collegial discussion within a multidisciplinary author team comprising anaesthesiologists, cardiologists, surgeons, and translational researchers. Although duplicate independent screening of identified records was not feasible owing to resource constraints, study selection and data extraction were performed by the lead authors (D.S.P., L.L.V., M.L.P.) and any disagreement regarding eligibility, interpretation, or weighting of evidence was resolved by collegial consensus, with adjudication by the senior authors (M.B., M.S.) when required. Evidence prioritisation followed pre-specified criteria favouring randomised controlled trials, meta-analyses, and contemporary clinical guidelines, with explicit acknowledgement of methodological limitations and conflicting findings throughout the synthesis.

Additional limitations include the predominant restriction to English-language publications, which may have omitted relevant non-English evidence, and the potential influence of publication bias toward positive studies. Substantial heterogeneity across populations, interventions, and outcome definitions limits definitive conclusions. The rapidly evolving nature of the field—particularly regarding artificial intelligence applications and wearable technologies—means that some included evidence may be superseded by emerging data. Despite these limitations, the present synthesis integrates contemporary evidence with current clinical guidelines to inform practice and identify research priorities while transparently signalling areas of uncertainty and active investigation.

## 3. Pathophysiology and Risk Stratification

### 3.1. Mechanistic Underpinnings of Perioperative Arrhythmias

Perioperative arrhythmias arise from a complex interaction of mechanical, metabolic, inflammatory, and autonomic influences, frequently superimposed on underlying structural heart disease. Surgical trauma—particularly during cardiac interventions—provokes ischaemia–reperfusion injury that disrupts mitochondrial respiration in atrial myocytes, generates reactive oxygen species, and alters calcium homeostasis, all potent arrhythmogenic triggers [2,17]. Oxidative stress, including heightened atrial peroxynitrite levels, has been identified as a central contributor to POAF [2]. Systemic inflammation driven by cardiopulmonary bypass or tissue damage triggers cytokine cascades that foster conduction abnormalities and facilitate reentrant circuits, with a temporal association between postoperative peaks in inflammatory markers and arrhythmia onset; genetic predisposition has been hypothesised to amplify this inflammatory response and heighten susceptibility to POAF, as suggested by the association between the −174 G/C interleukin-6 promoter polymorphism, postoperative IL-6 levels, and POAF [18].

Autonomic imbalance—enhanced sympathetic activity and disrupted sympathovagal balance, often intensified by perioperative stress and catecholamine administration—promotes triggered activity and reentry, particularly within the atria [2]. This mechanism underlies the efficacy of beta-blockers in reducing perioperative AF [2,19]. Additional stressors, including hypoxaemia, electrolyte imbalances (especially hypokalaemia and hypomagnesaemia), and metabolic disturbances, further impair cardiac electrophysiology [9,20], and correction of these conditions is fundamental to perioperative arrhythmia management [2]. In summary, perioperative arrhythmias are mechanistically driven by oxidative stress, inflammation, autonomic imbalance, and transient metabolic or mechanical stressors that interact synergistically in susceptible individuals.

### 3.2. Risk Stratification

Contemporary perioperative management emphasises identification of high-risk patients to enable targeted, individualised prophylaxis. Risk stratification rests on patient-specific and procedural determinants, integrated with established cardiovascular risk scores. Several validated models predict POAF by incorporating advanced age, prior AF, elevated preoperative heart rate, extent of surgery, and comorbidities such as heart failure, hypertension, diabetes, and prior stroke. The SCA and EACTA recommend classifying patients into “standard” and “higher” risk categories accordingly, although no universally validated score exists specifically for perioperative AF after cardiac surgery [21,22]. The CHADS_2_ and CHA_2_DS_2_-VASc scores, although developed for stroke risk in non-surgical AF, retain utility in predicting new-onset perioperative AF [23,24], while the perioperative-specific POAF score—age, prior AF, COPD, valve surgery, and renal function—achieves a C-statistic of 0.77 in validation cohorts [8]. Machine-learning models may extend this performance, capturing non-linear relationships among predictors; a systematic review and meta-analysis reported a moderate pooled AUC of 0.84 (95% CI 0.80–0.87) for predicting POAF after coronary artery bypass grafting, exceeding conventional scores but limited by substantial heterogeneity and risk of bias that mandate external validation [11].

Biomarkers and electrocardiographic parameters increasingly complement clinical scores. NT-proBNP exceeding 400 pg/mL is associated with a three-fold increased risk of POAF after non-cardiac surgery [5], and high-sensitivity C-reactive protein (hs-CRP) correlates with arrhythmia risk, reflecting the inflammatory component of arrhythmogenesis [25]. Among ECG markers, P-wave duration exceeding 120 ms identifies atrial conduction abnormalities predisposing to AF [26,27], and ambulatory detection of frequent premature atrial contractions has been associated with increased risk of atrial fibrillation in observational cohorts [26,27]. For ventricular arrhythmias, asymptomatic premature ventricular contractions and non-sustained ventricular tachycardia generally do not increase perioperative cardiac risk; however, frequent or symptomatic ventricular arrhythmias—particularly with structural heart disease—warrant careful assessment and possible monitoring [28,29]. A preoperative 12-lead ECG is reasonable in patients with arrhythmia history, structural heart disease, or higher-risk surgery [30], and continuous ECG monitoring may be indicated in high-risk patients, including those with channelopathies, heart failure, or prior significant arrhythmias [7].

Although primarily aimed at ischaemic rather than arrhythmic events, perioperative cardiovascular risk assessment shares several predictors and provides complementary context: validated indices (RCRI [31], NSQIP [32]) and biomarkers (BNP/NT-proBNP, troponin) estimate the risk of major adverse cardiovascular events and inform monitoring intensity, with the 2022 ESC [33] and 2024 AHA/ACC [34] perioperative guidelines offering updated frameworks.

Two underappreciated modifiers deserve explicit integration. Women carry a disproportionately high relative risk of POAF despite their lower baseline AF prevalence and show differential drug metabolism with greater susceptibility to drug-induced QT prolongation and torsade de pointes than men [35,36]; sex differences in responses to rate- and rhythm-control and in thromboembolic risk at equivalent CHA_2_DS_2_-VASc scores carry direct implications for pharmacological management and anticoagulation thresholds [35]. Frailty, in turn, is an increasingly relevant modifier as ageing, multimorbid patients constitute a growing surgical population: integrating frailty indices such as the modified Frailty Index with cardiovascular scores such as the RCRI may better capture the elevated arrhythmic risk that standard scores underestimate and may guide monitoring and prophylaxis [37].

The integration of these patient-specific modifiers—sex, frailty, biomarkers, and AI-derived risk—into a unified, individualised stratification framework represents the core of a personalised perioperative approach and the conceptual axis of the present review.

## 4. Anaesthetic Considerations and Drug Interactions

Anaesthetic agents influence cardiac electrophysiology through autonomic modulation, direct ion-channel effects, and pharmacological interactions, altering myocardial depolarisation and repolarisation—changes often reflected in the QT interval. QT prolongation increases the heterogeneity of ventricular repolarisation and the risk of malignant arrhythmias, including torsade de pointes. QT dispersion (QTd), the difference between maximum and minimum QT across the 12-lead ECG, is an indirect marker of repolarisation inhomogeneity and arrhythmogenic potential [6,7]. The perioperative setting amplifies this risk through sympathetic stimulation, surgical stress, electrolyte disturbances, and concurrent exposure to multiple QT-prolonging drugs.

### 4.1. Volatile Anaesthetic Agents and Cardiac Repolarisation

Halothane may affect repolarisation by inhibiting the human ether-related gene (HERG) potassium current and transiently increasing repolarisation dispersion, yet clinical studies show minimal QT prolongation and low arrhythmogenic risk except where repolarisation is already abnormal [6]. Halogenated agents—sevoflurane, isoflurane, and desflurane—can prolong the QT interval and increase QT dispersion: administration at one minimum alveolar concentration (MAC) produces significant QTc prolongation shortly after the target concentration is reached, indicating a dose-dependent effect largely independent of the specific agent [7]. Inter-agent differences are modest, although desflurane has been associated with more pronounced QTc prolongation during induction, likely reflecting its sympathomimetic properties; rapid increases in desflurane concentration may further augment sympathetic activation and the risk of tachyarrhythmias, particularly in coronary or structural heart disease [6,7]. Mechanistically, sevoflurane inhibits the rapid and slow delayed-rectifier potassium currents (IKr, IKs); isoflurane primarily restricts the slow component; and halothane blocks HERG channels, potentially increasing transmural dispersion of repolarisation [6,7]. Although modern volatile agents are generally of low arrhythmogenicity, they may precipitate life-threatening arrhythmias in congenital long QT syndrome (LQTS) [6].

### 4.2. Intravenous Anaesthetic Agents

Propofol generally preserves haemodynamic stability and carries a low proarrhythmic risk and may exert antiarrhythmic effects through calcium-channel inhibition and sympathetic attenuation; at high concentrations however, it can prolong atrioventricular (AV) conduction, with rare reports of bradycardia, torsade de pointes, or ventricular fibrillation [28]. Etomidate is often preferred in cardiovascular disease owing to its minimal haemodynamic effects and negligible influence on ventricular repolarisation and QT duration [29]. Ketamine, by contrast, has sympathomimetic properties that raise heart rate and arterial pressure, making it less suitable in high-risk patients such as those with LQTS [6].

Among the α_2_-agonists, dexmedetomidine has attracted particular interest for arrhythmia prevention through sympatholysis and attenuation of the inflammatory response. Although the pivotal DECADE randomised trial did not demonstrate a significant reduction in postoperative atrial fibrillation [30], a subsequent systematic review and meta-analysis of 18 randomised trials (2933 patients) found that perioperative dexmedetomidine significantly reduced POAF after cardiac surgery (odds ratio 0.82, 95% CI 0.69–0.98), without affecting stroke or mortality [38]. Notably, this benefit was concentrated in specific subgroups—patients undergoing coronary artery bypass grafting (CABG; odds ratio 0.65, 95% CI 0.46–0.91) and younger and female patients—illustrating how the cardioprotective effect of dexmedetomidine is best understood as patient-dependent rather than uniform [38].

### 4.3. Local Anaesthetics and Cardiac Electrophysiology

Local anaesthetics act primarily by blocking voltage-gated sodium channels in nerve fibres; after systemic absorption or inadvertent intravascular injection they may also inhibit cardiac sodium (INa), L-type calcium (ICa-L), and repolarising potassium currents [6]. Bupivacaine carries the highest cardiotoxic potential—toxic concentrations affect sodium, calcium, and potassium (Ito, IKr) currents, producing AV block, ventricular ectopy, QRS widening, and potentially fatal ventricular fibrillation, with cardiovascular toxicity reported up to four times more frequently than with lidocaine [6]. Lidocaine, a class Ib agent, stabilises conduction through inactivated-state sodium-channel binding [6]. Ropivacaine and levobupivacaine were developed to reduce cardiotoxicity: ropivacaine may be a safer choice in conduction abnormalities, whereas levobupivacaine has occasionally been linked to QT prolongation [6]. Lipid emulsion therapy remains the cornerstone of management for local anaesthetic systemic toxicity (LAST) with arrhythmic manifestations [39].

### 4.4. Perioperative Factors Influencing Cardiac Repolarisation

Direct laryngoscopy and tracheal intubation are potent sympathetic stimuli that increase heart rate, arterial pressure, and QT duration, particularly in underlying cardiac disease; short-acting β-blockers such as landiolol attenuate intubation-related QT prolongation [6]. Regional anaesthesia also modulates repolarisation: high thoracic epidural blockade (T1–T4) may shorten the QT interval by reducing cardiac sympathetic tone, whereas lower blockade may provoke compensatory sympathetic activation and QT prolongation [6].

### 4.5. Critical Drug Interactions

Perioperative polypharmacy carries substantial potential for rhythm-altering interactions. Amiodarone potentiates the cardiac effects of volatile agents, with the risk of profound bradycardia and hypotension, warranting a reduction in volatile dosing and vigilant monitoring [40]. Beta-blockers may produce exaggerated negative inotropic and chronotropic effects with volatile agents and combined with propofol may cause marked hypotension [41]. Non-dihydropyridine calcium-channel blockers (verapamil, diltiazem) may exacerbate negative inotropy and enhance neuromuscular blockade [42]. Digoxin’s narrow therapeutic index becomes critical perioperatively, since hypokalaemia and hypomagnesaemia may precipitate toxicity [16]. Antiemetics and first-generation antihistamines may prolong the QT interval, warranting caution in LQTS or with other QT-active drugs [43]. Recognising these interactions allows clinicians to anticipate arrhythmic complications through judicious agent selection, dose adjustment, and enhanced monitoring. More broadly, the choice of anaesthetic technique is itself an individualised decision: agent selection should be tailored to the patient’s electrophysiological profile—favouring repolarisation-neutral agents in LQTS, haemodynamically stable agents in structural heart disease, and conduction-sparing local anaesthetics where conduction is impaired. Pharmacological management can thus be conceptualised as two complementary domains—preventive strategies targeting substrate modification and acute therapies for rate or rhythm control (Table 1 and Table 2).

### 4.6. Non-Antiarrhythmic QT-Prolonging Drugs and Torsade de Pointes Risk

Beyond anaesthetic agents and antiarrhythmics, the perioperative and critical-care environment routinely exposes patients to numerous non-cardiac drugs with recognised QT-prolonging potential, whose cumulative contribution is easily underestimated. Frequently implicated classes include macrolide (azithromycin, clarithromycin, erythromycin) and fluoroquinolone (moxifloxacin, levofloxacin, ciprofloxacin) antibiotics, azole antifungals (fluconazole, voriconazole), butyrophenone and other antipsychotics (haloperidol, droperidol), selected antidepressants (citalopram, escitalopram, tricyclics), methadone, and the antiemetics (notably ondansetron and droperidol) and first-generation antihistamines already noted [43,44]. The AZCERT CredibleMeds QTdrugs list provides a continuously updated, evidence-graded catalogue of these agents and serves as a practical bedside reference [45].

Crucially, torsade de pointes and other malignant ventricular arrhythmias rarely follow a single isolated exposure. They far more often reflect the convergence of several coexisting perioperative risk factors—baseline or congenital QT prolongation, female sex, structural heart disease, bradycardia, hepatic or renal impairment with reduced drug clearance, sepsis, hypokalaemia and hypomagnesaemia, volatile anaesthetics, and, above all, the concurrent use of two or more QT-active drugs or of agents that inhibit their metabolism, such as cytochrome P450 3A4 (CYP3A4) inhibition by clarithromycin or azole antifungals [43,44]. This cumulative, multi-hit model is operationalised by validated tools such as the Tisdale risk score, which integrates age, sex, serum electrolytes, admission diagnosis, baseline QTc, and the number of QT-prolonging drugs to stratify the probability of QTc exceeding 500 ms [46].

Effective mitigation therefore rests on anticipation and layered precautions rather than blanket avoidance of any single agent (Table 3): obtaining a baseline 12-lead ECG and QTc in high-risk patients; correcting and maintaining serum potassium and magnesium within the normal range; avoiding unnecessary combinations of QT-prolonging drugs and screening for pharmacokinetic interactions; adjusting doses in renal or hepatic dysfunction; substituting lower-risk alternatives where feasible; and instituting continuous ECG telemetry with serial QTc monitoring when high-risk combinations are unavoidable—prompting withdrawal of the offending agent when QTc exceeds 500 ms or rises by more than 60 ms from baseline [44,46].

## 5. Intraoperative Management Strategies

### 5.1. Evolution of Monitoring Approaches

Intraoperative arrhythmia detection has evolved well beyond basic electrocardiography. Continuous ECG monitoring remains the cornerstone, with modern systems incorporating multi-lead analysis, automated arrhythmia detection, and ST-segment trending that improve sensitivity for transient ischaemia-related events. Continuous ECG monitoring is recommended for all patients undergoing anaesthesia, with additional ischaemia monitoring in those at increased cardiovascular risk [47]. Haemodynamic monitoring provides complementary information, distinguishing electrically evident but clinically insignificant arrhythmias from those causing haemodynamic compromise; continuous arterial pressure monitoring enables beat-to-beat assessment, and escalation to invasive monitoring may be considered in selected high-risk patients within a risk-adapted framework.

Advanced haemodynamic systems—pulse-contour analysis and noninvasive cardiac output (CO) monitoring—may inform volume responsiveness, but their accuracy is significantly reduced in the presence of arrhythmias [48]. Pulmonary artery catheterisation offers comprehensive assessment in selected settings but is not supported for routine use [49]. Transoesophageal echocardiography (TEE) is a key adjunct in haemodynamic instability or suspected structural abnormalities, providing real-time assessment of ventricular function, preload, and regional wall motion [50], although its evidence base is largely observational and operator-dependent. Contemporary guidelines consistently advocate a stepwise, risk-adapted approach in which monitoring intensity is tailored to patient- and procedure-specific risk [33,34,47]; no single modality reduces major cardiovascular outcomes when applied indiscriminately, supporting a selective, individualised strategy.

Emerging AI systems integrating continuous arterial-waveform data to predict haemodynamic instability are promising but remain preliminary and methodologically debated [51].

### 5.2. Prophylactic Interventions

Beta-blockade is the most extensively studied prophylactic intervention, with systematic reviews indicating an approximately 30% reduction in POAF, particularly in cardiac surgery [52]. Timing is important: initiation at least 24 h preoperatively with postoperative continuation maximises efficacy [53]. This evidence pertains specifically to POAF prophylaxis in cardiac surgery and should not be extrapolated to de novo perioperative beta-blockade in non-cardiac surgery, where the POISE trial cautioned that high-dose initiation increases stroke and mortality despite reducing atrial fibrillation [54].

Magnesium supplementation provides modest protection against perioperative supraventricular arrhythmias. Meta-analyses indicate that intravenous magnesium (typically 2 g) reduces POAF incidence by approximately 23% in cardiac surgery through calcium-channel antagonism, potassium-channel modulation, and anti-inflammatory effects [55,56,57]; the optimal regimen combines pre- and post-bypass administration to maintain serum magnesium within the normal range.

Amiodarone prophylaxis is effective in high-risk populations: a loading dose of 300 mg followed by continuous infusion reduced POAF from 33% to 22% in a systematic review of randomised trials [58]; however, bradycardia, hypotension, and pulmonary toxicity warrant restriction to the highest-risk patients. Active normothermia confers no additional cardiovascular benefit over mild controlled hypothermia: although older, smaller studies linked intraoperative hypothermia to excess cardiac events driven partly by ventricular tachycardia [59], the large PROTECT trial showed that targeting 37.0 °C did not reduce major cardiovascular outcomes compared with 35.5 °C, indicating that a core temperature of at least 35.5 °C is sufficient and safe [60].

### 5.3. Intraoperative Management of Specific Arrhythmias

Management begins, in all cases, with rapid identification and correction of reversible precipitants—hypoxaemia, hypercapnia, electrolyte disturbances, myocardial ischaemia, hypovolaemia, inadequate anaesthetic depth, and surgical stimulation—since perioperative arrhythmias are frequently secondary to physiological derangement rather than primary electrical disorders [61].

Supraventricular tachyarrhythmias require differentiation between unstable rhythms needing immediate intervention and stable rhythms permitting a conservative approach. For stable regular narrow-complex tachycardias, vagal manoeuvres may be attempted, followed by adenosine (6–12 mg IV bolus) for diagnosis and potential termination [62]; if these fail or are contraindicated, short-acting AV-nodal blocking agents may be considered in selected stable patients [16]. Atrial fibrillation or flutter with rapid ventricular response is managed according to haemodynamic status: instability mandates immediate synchronised cardioversion [63], whereas in stable patients management prioritises correction of triggers and rate control, the preferred acute strategy [16]. Beta-blockers (e.g., esmolol) are generally first-line, with non-dihydropyridine calcium-channel blockers as alternatives in preserved ventricular function [16]; a lenient rate-control target (<110 bpm) is acceptable in many patients, individualised to clinical context and symptom burden [61]. Intravenous amiodarone or digoxin may be considered in impaired ventricular function or inadequate response to first-line agents [16], and rate- and rhythm-control strategies may be combined or applied sequentially [16].

Ventricular arrhythmias require immediate assessment. Isolated premature ventricular complexes (PVCs) or short runs of non-sustained ventricular tachycardia (NSVT) do not generally require specific therapy but should prompt correction of reversible factors. Sustained ventricular tachycardia (VT) with a pulse and instability requires immediate synchronised cardioversion, whereas pulseless VT or ventricular fibrillation (VF) mandates defibrillation and advanced cardiac life support (ACLS) [63]. In stable wide-complex tachycardia, amiodarone or procainamide may be considered [63]. Polymorphic VT—particularly torsade de pointes—is frequently associated with QT prolongation, electrolyte disturbances, and drug effects; management includes intravenous magnesium and correction of precipitants, with immediate defibrillation if unstable [63]. Bradyarrhythmias, commonly related to vagal tone, anaesthetic agents, hypoxaemia, or surgical stimuli, are managed by correcting reversible causes and adjusting anaesthetic depth; atropine (0.5 mg IV, repeatable every 3–5 min to a maximum of 3 mg) is appropriate, while persistent compromise necessitates temporary pacing [64].

## 6. Postoperative Critical-Care Approaches

### 6.1. Rate Versus Rhythm Control Strategies

Management of postoperative atrial fibrillation requires a strategic choice between rate control and rhythm restoration. The landmark CTSN trial [65] tested whether rhythm control—amiodarone and/or direct-current cardioversion—offered any advantage over rate control (beta-blockers, calcium-channel blockers, or digoxin) in haemodynamically stable patients with new-onset POAF after cardiac surgery and found no significant difference in hospital length of stay, morbidity, or mortality [65]. In non-surgical AF, the RACE II trial had already shown that a lenient resting heart rate target (<110 bpm) was non-inferior to a strict target (<80 bpm) for cardiovascular outcomes [61]. Together these data have shifted perioperative practice toward initial rate control in stable patients.

Contemporary management has nonetheless become more individualised. Current ACC/AHA guidance accepts both rate control (preferentially beta-blockers, or non-dihydropyridine calcium-channel blockers if beta-blockers are contraindicated) and rhythm control as initial strategies, selected according to symptoms, haemodynamic consequences, and clinical judgement [16]. Haemodynamically unstable or poorly tolerated AF warrants immediate cardioversion with antiarrhythmic therapy, with left atrial appendage imaging before cardioversion if AF has persisted beyond 48 h without anticoagulation [16]. In critically ill patients, aggressive management of underlying triggers—sepsis, pain, anaemia, and electrolyte disturbances—is the cornerstone of AF management [66]. For acute rate control, intravenous beta-blockers (metoprolol, esmolol) or non-dihydropyridine calcium-channel blockers (diltiazem, verapamil) are used, with beta-blockers preferred in coronary disease or heart failure and calcium-channel blockers avoided in moderate-to-severe left ventricular systolic dysfunction [16]; ultra-short-acting agents such as esmolol and landiolol offer rapidly titratable, reversible control [67]. Digoxin is an adjunct when first-line agents fail or are contraindicated, despite slow onset and limited efficacy during exertion [16], and intravenous magnesium is a reasonable adjunct to standard rate control [16].

Spontaneous conversion to sinus rhythm occurs in a substantial proportion of POAF cases—earlier cardiac surgery cohorts reported high rates of spontaneous resolution within the first postoperative days [68,69]—although conversion may continue over several weeks, so the frequently cited 24–48 h window likely underestimates the full conversion period [65]. These data support conservative management in stable patients, with rhythm assessment and possible delayed cardioversion reserved for those with persistent AF at 30–60-day follow-up [16]. POAF is nonetheless associated with significantly increased short-term risk—a meta-analysis of 35 studies reported a 62% higher early stroke risk (OR 1.62) and a 44% higher early mortality (OR 1.44)—as well as a 37% higher long-term stroke risk (HR 1.37) and excess long-term mortality, underscoring the importance of appropriate anticoagulation and surveillance [34,70,71].

### 6.2. Anticoagulation Considerations

Anticoagulation requires careful balancing of thromboembolic and bleeding risks across the perioperative continuum. For patients on chronic anticoagulation undergoing elective surgery, contemporary evidence supports temporary cessation without bridging in most cases [72,73]: the BRIDGE trial showed that bridging with low-molecular-weight heparin increased major bleeding (3.2% vs. 1.3%) without reducing thromboembolism (0.4% vs. 0.3%), establishing simple interruption as preferred for patients without recent stroke, transient ischaemic attack, or mechanical valves [73]. Direct oral anticoagulants (DOACs) have simplified management, with the PAUSE study (3007 patients) validating a standardised interruption protocol associated with low rates of major bleeding (<2%: 1.35%, 0.90%, and 1.85% in the apixaban, dabigatran, and rivaroxaban cohorts, respectively) and arterial thromboembolism (<1%: 0.16–0.60%) [74,75].

Postoperative decisions are more complex. New-onset POAF occurs in 3–16% of non-cardiac and up to 40% of cardiac surgery patients [1,2,76], conferring a greater than two-fold increased risk of in-hospital ischaemic stroke [66,77]. Initiation should be guided by CHA_2_DS_2_-VASc stratification, balancing stroke prevention against bleeding risk; current recommendations support therapeutic anticoagulation within 12–48 h of cardiac surgery once haemostasis is adequate, for a minimum of four weeks [17,34,66,77]. This recommendation rests primarily on expert consensus and observational data rather than high-quality randomised evidence—the scarcity of prospective trials on optimal timing in postoperative POAF makes this a domain of genuine clinical uncertainty, explicitly acknowledged in the 2024 AHA/ACC perioperative guideline [34]. In sepsis-associated AF, CHA_2_DS_2_-VASc poorly predicts stroke risk and routine acute anticoagulation has shown no benefit while increasing bleeding, making individualised, patient-specific assessment essential [66]—a clear example of where generic risk scores must yield to personalised judgement.

A distinct and clinically pressing question concerns the duration of anticoagulation once the initial postoperative period has elapsed—particularly after cardiac surgery, where POAF has traditionally been regarded as a transient, reactive phenomenon that resolves as the patient recovers. Whether a single, self-terminating episode of POAF carries the same long-term thromboembolic risk as spontaneous clinical AF and therefore warrants indefinite anticoagulation remains unresolved. On the one hand, new-onset POAF after cardiac surgery is associated with a durably increased risk of late stroke and death (long-term stroke hazard ratio approximately 1.37), suggesting that it may unmask a pre-existing atrial substrate rather than represent a purely benign electrical disturbance [70,71]. On the other hand, a substantial proportion of patients revert to sinus rhythm and never again manifest documented AF, so that indiscriminate lifelong anticoagulation risks bleeding without commensurate benefit [78]. Contemporary observational data reinforce this uncertainty: in new-onset POAF after coronary artery bypass grafting, oral anticoagulation has not been shown to reduce thromboembolic events at 60 days or at one year—despite a signal towards lower early all-cause mortality—so that the net benefit of longer-term therapy remains unproven [79]. Accordingly, the 2023 ACC/AHA/ACCP/HRS guideline recommends individualised, CHA_2_DS_2_-VASc-guided anticoagulation for approximately 60 days after surgery, coupled with explicit reassessment of both AF recurrence and the continued indication for anticoagulation thereafter, rather than automatic lifelong treatment [16]. Structured rhythm surveillance—increasingly feasible with the ambulatory and wearable technologies discussed below—is therefore central to identifying the subset with recurrent paroxysmal or persistent AF who stand to benefit from sustained anticoagulation, while sparing those with a single, truly transient episode. The randomised PACES trial (Anticoagulation for New-Onset Post-Operative Atrial Fibrillation After CABG; NCT04045665) is expected to place these decisions on firmer evidentiary ground [78].

For resumption of chronic anticoagulation, timing depends on procedural bleeding risk and haemostasis: DOACs may restart as early as 24 h after low-bleeding-risk procedures but should be delayed 48–72 h after high-risk procedures, while warfarin can usually restart on the evening of surgery given its delayed effect [74]. Postoperative bridging is generally not recommended, as it increases bleeding without reducing thromboembolism [72,73]. When bleeding complications occur, management includes discontinuation of anticoagulants, resuscitation, correction of hypothermia and acidosis, and reversal agents—prothrombin complex concentrates for warfarin or factor Xa inhibitors, and idarucizumab for dabigatran [80].

### 6.3. Electrolyte Management

Electrolyte disturbances are critical, modifiable risk factors for perioperative arrhythmias. Hypokalaemia is particularly prevalent and arrhythmogenic, occurring in 35.7% of patients presenting with ventricular tachycardia or fibrillation, with severe hypokalaemia (potassium < 3.0 mEq/L) in 13.6% [81]. In critically ill patients, potassium below 3.5 mEq/L is associated with increased hazard for clinically significant arrhythmias (HR 1.23–1.26), rising as hypokalaemia worsens [82]. However, the 2024 TIGHT K trial demonstrated that maintaining potassium above 3.6 mEq/L is non-inferior to maintaining it above 4.5 mEq/L for preventing AF after cardiac surgery, indicating that routine tight control—and the associated supplementation burden—is unnecessary and potentially harmful [83]. Current guidance recommends maintaining potassium above 4.0 mEq/L in documented life-threatening ventricular arrhythmias or acute myocardial infarction, although observational data suggest ventricular arrhythmia rates rise mainly below 3.0 or above 5.0 mEq/L [83].

Magnesium plays a complementary role: it is first-line therapy for torsade de pointes and facilitates potassium repletion when both are depleted [63]. A 2025 quasi-experimental study using a regression-discontinuity design found no significant benefit of routine magnesium supplementation for preventing tachyarrhythmias in critically ill patients [84]; this should be read as hypothesis-generating evidence requiring prospective confirmation rather than practice-changing data, and the established recommendation for intravenous magnesium as first-line therapy in torsade de pointes is unaffected [83]. Observational MIMIC-IV analyses have linked both hypo- and hypercalcaemia and a U-shaped phosphate relationship to increased mortality in critically ill AF patients [85,86]; these single-centre retrospective findings carry substantial methodological limitations and should be regarded as supplementary and hypothesis-generating rather than a basis for definitive practice change.

Practically, repletion should prioritise correction of severe hypokalaemia (potassium 3.0–3.5 mEq/L), replete magnesium before or with potassium when both are deficient, maintain normal-range electrolytes in patients receiving QT-prolonging antiarrhythmics, and avoid overly aggressive supplementation that adds cost and patient burden without proven benefit [87,88]. Risk factors strongly associated with severe postoperative hypokalaemia—gastrointestinal illness and recent diuretic dose increases—represent opportunities for early, individualised intervention [81].

## 7. Special Populations

The perioperative care of patients with cardiac implantable electronic devices and inherited arrhythmia syndromes epitomises a genotype- and phenotype-driven, individualised approach, in which management is dictated less by the procedure than by the patient’s specific electrophysiological substrate.

### 7.1. Patients with Cardiac Implantable Electronic Devices

Patients with cardiac implantable electronic devices (CIEDs)—pacemakers and implantable cardioverter-defibrillators (ICDs)—are increasingly encountered perioperatively and pose specific risks of arrhythmia and device malfunction. Electromagnetic interference (EMI), particularly from monopolar electrocautery, is the most relevant trigger of device dysfunction, potentially causing inappropriate inhibition of pacing in pacemaker-dependent patients or unintended ICD therapy, with possible haemodynamic consequences [89,90]. Anaesthesia-related autonomic fluctuations, hypoxia, and electrolyte imbalances may compound this risk.

A comprehensive preoperative evaluation is essential and should include device interrogation, assessment of pacing dependency and underlying rhythm, the indication for implantation, and identification of manufacturer-specific characteristics that may influence perioperative behaviour [89,91]. Management is individualised: in pacemaker-dependent patients, reprogramming to an asynchronous mode or magnet application prevents oversensing, whereas ICD antitachycardia therapies are generally suspended during surgery to avoid inappropriate shocks [89,90]. The magnet response differs by device type and must be understood in advance—over a pacemaker a magnet generally induces asynchronous pacing, whereas over an ICD it suspends antitachycardia therapies but does not affect pacing—so reliance on magnet application without prior device characterisation may be unsafe [90]. Intraoperatively, continuous ECG and haemodynamic monitoring is mandatory, and EMI-minimising strategies—bipolar cautery, short energy bursts, and appropriate return-electrode placement—should be implemented systematically [89,90]; external defibrillation and temporary pacing must be immediately available, particularly in pacing-dependent patients. Postoperative interrogation should be performed promptly to restore baseline settings and detect lead dysfunction, battery issues, or inappropriate therapies [89,90]. Multidisciplinary collaboration between cardiologists, anaesthesiologists, and surgeons remains essential [89,91].

### 7.2. Patients with Inherited Arrhythmia Syndromes

Patients with inherited cardiac channelopathies—long QT syndrome (LQTS), Brugada syndrome, catecholaminergic polymorphic ventricular tachycardia (CPVT), and short QT syndrome (SQTS)—are at high risk of perioperative malignant arrhythmias owing to intrinsic electrical instability and sensitivity to external triggers. Perioperative events are typically precipitated by the interplay of autonomic fluctuations, electrolyte disturbances, and drugs that modulate cardiac ion channels [92,93]. LQTS, the most extensively studied, is characterised by delayed repolarisation predisposing to torsade de pointes, often triggered by sympathetic activation, anaesthetic agents, or QT-prolonging medications [93]; management centres on strict avoidance of QT-prolonging drugs, maintenance of potassium and magnesium balance, and attenuation of adrenergic surges through adequate analgesia and anxiolysis [93].

Brugada syndrome carries a risk of life-threatening ventricular arrhythmias from sodium-channel dysfunction, with perioperative triggers including fever, vagal predominance, and exposure to specific anaesthetic or antiarrhythmic agents that may exacerbate the characteristic ST-segment abnormalities [92]. Sodium-channel-blocking local anaesthetics warrant particular caution, and an up-to-date, consensus-based list of drugs to avoid is maintained at brugadadrugs.org [94]. Careful drug selection and continuous ECG monitoring with attention to the right precordial leads (V1–V3) are therefore essential. CPVT is characterised by adrenergically mediated polymorphic ventricular arrhythmias in structurally normal hearts, triggered by emotional or physical stress [95]; the perioperative period is hazardous owing to surgical catecholamine surges, and management is directed at minimising sympathetic activation, with β-blockade as the cornerstone [95]. Dexmedetomidine has been proposed as a sympatholytic adjunct, but this is a mechanistic extrapolation with no studies specifically evaluating it in perioperative CPVT and should be regarded as speculative; more broadly, perioperative CPVT management derives largely from case reports and physiological reasoning rather than controlled evidence. Even with a normal baseline ECG, these patients remain at high risk, underscoring the need for a high index of suspicion.

SQTS is associated with abbreviated repolarisation and increased susceptibility to atrial and ventricular arrhythmias, including sudden cardiac death [93]. Causative variants predominantly involve cardiac potassium-channel genes (KCNH2, KCNQ1, KCNJ2), and long-term follow-up confirms a high rate of sudden cardiac death and ventricular fibrillation in affected families, with arrhythmic events occurring in a substantial proportion of patients during prolonged observation [96,97,98]. Given its rarity, perioperative evidence is limited and management is extrapolated from general channelopathy principles—avoidance of proarrhythmic triggers and immediate readiness for defibrillation. Overall, perioperative management of channelopathies requires an individualised, mechanism-based approach combining meticulous drug selection, strict autonomic control, and continuous monitoring; the evidence base remains predominantly observational—especially for CPVT and SQTS—highlighting the need for prospective studies and standardised protocols.

## 8. Emerging Technologies and Future Directions

### 8.1. Artificial Intelligence Applications

By integrating large volumes of heterogeneous data—clinical variables, comorbidities, and potentially biomarker- and ECG-derived information—artificial intelligence (AI) and machine-learning (ML) algorithms have shown predictive performance superior to traditional risk scores. In particular, ML models can capture complex, non-linear relationships among variables that conventional tools such as the CHA_2_DS_2_-VASc score, whose discrimination remains moderate [99], do not represent. In perioperative and AF populations, machine-learning algorithms have achieved moderate discrimination for adverse cardiovascular events (pooled AUC 0.84), supporting a potential role in more precise, individualised risk stratification, although current evidence is heterogeneous and at high risk of bias [11]. Beyond prediction, convolutional neural networks analysing continuous ECG can detect subtle patterns presaging arrhythmias before conventional algorithms, potentially enabling pre-emptive intervention [12,13,100,101]. Despite this promise, barriers to implementation persist: regulatory uncertainty, algorithmic opacity (“black-box” concerns), the complexity of electronic health record (EHR) integration, and the need for validation across diverse populations before routine clinical adoption.

### 8.2. Advanced Monitoring Technologies

Contemporary patch-based ECG monitors enable up to 14-day continuous recording with high adherence [102]. Consumer smartwatch photoplethysmography (PPG) algorithms can identify atrial fibrillation: the Apple Heart Study, a large single-arm screening study (~419,000 participants), reported a positive predictive value of 0.84 (95% CI 0.76–0.92) for an irregular-pulse notification against simultaneous ECG patch monitoring—a screening yield rather than formal sensitivity and specificity [103]. The mAFA-II cluster-randomised trial (3324 patients) evaluated a smartphone-based integrated-care pathway and reported a significant reduction in the composite outcome of ischaemic stroke or systemic thromboembolism, all-cause death, and rehospitalisation (hazard ratio 0.39, 95% CI 0.22–0.67), driven largely by fewer rehospitalisations [14], supporting structured post-discharge monitoring in high-risk surgical patients. Implantable loop recorders (ILRs) provide up to three years of continuous surveillance: in CRYSTAL-AF, ILR monitoring detected AF in 12.4% of patients versus 2.0% with conventional monitoring at twelve months [104]. Notably, CRYSTAL-AF enrolled patients with cryptogenic stroke rather than surgical patients, so its findings cannot be directly extrapolated to perioperative populations; nonetheless, the superior detection yield of continuous long-term monitoring over intermittent external approaches is well established. Integration of data across these sources requires robust platforms addressing security, privacy, and regulatory compliance.

### 8.3. Novel Therapeutic Approaches

Agents targeting atrial-specific ion channels—including IKACh blockers and vernakalant—aim to reduce ventricular proarrhythmic risk. Budiodarone, combining potassium-channel blockade with anti-inflammatory properties, produced a dose-dependent reduction in AF burden versus placebo in the phase II PASCAL trial (72 patients), with reductions of 54.4% (400 mg twice daily) and 75% (600 mg twice daily) and no drug-related serious adverse events [15]; perioperative applications remain investigational. Low-level transcutaneous vagal nerve stimulation has shown promise: the TREAT-AF pilot randomised trial (54 cardiac surgery patients) reported a significant reduction in POAF versus sham [105], though confirmation in larger trials is required. Renal denervation may attenuate sympathetic drive, but perioperative trials are lacking. Among anti-inflammatory strategies, colchicine has shown clinically meaningful POAF reductions: the COPPS and COPPS-2 trials, with pooled cardiac-surgery analyses, reported an approximately 40–45% relative risk reduction at low cost [106,107], although gastrointestinal intolerance led to non-trivial discontinuation rates and tempered enthusiasm. Corticosteroids show inconsistent efficacy with concerning adverse effects, while targeted anti-cytokine therapies (interleukin-1 and interleukin-6 antagonists) remain investigational.

### 8.4. Future Research Priorities

The highest priority is the development and validation of personalised prevention strategies that integrate clinical variables with genomic data, biomarkers, and AI-based risk prediction [51,108]—the approach most aligned with precision perioperative care. This requires robust comparative-effectiveness studies validating and generalising AI models across diverse healthcare settings [108], randomised trials defining the optimal duration and intensity of post-discharge monitoring with accompanying cost-effectiveness analyses, and phase III perioperative trials of novel atrial-selective agents such as budiodarone [15]. Finally, prospective cohort studies are needed to clarify the long-term significance of perioperative arrhythmias—specifically whether transient events confer increased future cardiovascular risk.

## 9. Conclusions and Clinical Recommendations

Perioperative arrhythmias are increasingly preventable through the systematic integration of risk assessment, evidence-based prophylaxis, vigilant monitoring, and appropriate acute and chronic management, applied within an individualised, patient-tailored framework.

Systematic risk stratification combining clinical scores (CHA_2_DS_2_-VASc, POAF score), biomarkers (NT-proBNP, high-sensitivity C-reactive protein), and electrocardiographic parameters (P-wave duration) identifies high-risk patients, with machine-learning models showing moderate discrimination (pooled AUC 0.84) and potentially enabling more individualised management pending external validation [11]. Understanding agent-specific electrophysiological effects—the QT-prolonging properties of sevoflurane, the sympathomimetic effects of desflurane, and the relative neutrality of propofol—permits rational anaesthetic selection tailored to the patient’s electrophysiological profile, with anticipation of interactions involving antiarrhythmics, beta-blockers, and calcium-channel blockers.

Evidence-based prophylaxis includes perioperative beta-blockade initiated at least 24 h preoperatively (~30% POAF reduction in cardiac surgery) [52,53], magnesium supplementation (2 g IV; ~23% reduction) [55], selective amiodarone reserved for the highest-risk patients given its toxicity [58], dexmedetomidine (meta-analytic odds ratio 0.82, with benefit concentrated in CABG and younger and female patients) [38], and the emerging anti-inflammatory option colchicine (~40–45% reduction in cardiac surgery, COPPS/COPPS-2) [106,107]. Novel agents such as budiodarone show improved safety profiles pending perioperative validation [15].

Acute management should be context- and patient-specific: haemodynamically stable POAF favours rate control, with spontaneous conversion occurring in a substantial proportion of patients over hours to weeks [65]; haemodynamically significant arrhythmias require electrical cardioversion [63], and ventricular arrhythmias mandate ACLS-protocol intervention [63]. Postoperative care encompasses CHA_2_DS_2_-VASc-guided anticoagulation—with optimal timing and duration still to be defined by prospective randomised evidence—and electrolyte repletion guided by contemporary trials: potassium should be maintained above 3.6 mEq/L rather than at previously advocated high-normal thresholds (TIGHT K) [83], avoiding aggressive normalisation that adds risk and cost without benefit. Extended monitoring via wearable technologies enables detection of recurrent arrhythmias within integrated care pathways [14].

From a health-economic perspective, POAF substantially increases per-patient costs [4]; prophylactic beta-blockade and magnesium offer favourable cost-effectiveness, whereas universal amiodarone or extensive ILR deployment may be less sustainable, and AI-supported integrated monitoring shows promising cost-effectiveness in implementation studies [14]. Through multidisciplinary collaboration across anaesthesiology, cardiology, electrophysiology, and critical care—and through systematic quality improvement—the burden of perioperative arrhythmias can be progressively reduced. Optimal management transcends arrhythmia recognition, representing comprehensive, personalised cardiovascular care across the perioperative continuum.

## Figures and Tables

**Table 1:** Pharmacological strategies for prevention of perioperative arrhythmias.

Drug/Class	Mechanism	Perioperative Indication	Clinical Use	Benefits	Risks/Limitations	Level of Evidence
Beta-blockers (esmolol, metoprolol, landiolol)	β-adrenergic blockade; ↓ sympathetic tone, automaticity	Prevention of POAF (especially cardiac surgery)	Initiation ≥ 24 h preop and continuation postop in high-risk patients	~30% reduction in POAF; strong guideline support	Bradycardia, hypotension, bronchospasm; not for de novo use in non-cardiac surgery (POISE)	High (RCTs, meta-analyses, guidelines)
Amiodarone (prophylactic)	Multichannel blockade; prolongs refractoriness	Prevention of POAF in high-risk patients	Selected prophylaxis (not routine) in cardiac surgery	Reduces POAF incidence (33% → 22%)	Bradycardia, hypotension, QT prolongation, systemic toxicity	High–moderate (RCTs, meta-analyses)
Magnesium sulfate	Modulates Ca^2+^ influx; stabilises myocardium	Adjunct prevention of POAF	IV perioperative supplementation (pre/post-bypass)	Safe, low cost; ~23% POAF reduction	Limited benefit if normomagnesaemic; renal caution	Moderate (meta-analyses, heterogeneous RCTs)
Dexmedetomidine	α_2_-agonist; sympatholysis, anti-inflammatory	Prevention of POAF (cardiac surgery)	Intra/postoperative infusion in selected patients	~18% relative reduction (OR 0.82); benefit concentrated in CABG/younger/female subgroups	Bradycardia, hypotension	Moderate (meta-analysis of RCTs; pivotal RCT DECADE neutral)
Colchicine	Anti-inflammatory (IL-1 pathway)	Prevention of POAF (adjunct)	Selected cardiac surgery patients	~40–45% POAF reduction (COPPS/COPPS-2)	GI intolerance; non-trivial discontinuation	Moderate (RCTs, meta-analyses)
Electrolyte optimisation (K^+^, Mg^2+^)	Restores membrane stability	Universal preventive strategy	Maintain K^+^ > 3.6 mEq/L (TIGHT K); avoid severe hypoK/Mg	Reduces arrhythmogenic substrate	Overcorrection; avoid aggressive supplementation	Moderate (RCTs incl. TIGHT K)
Statins/anti-inflammatory strategies	Anti-inflammatory, pleiotropic	Investigational adjunct	Not routine	Possible reduction in POAF	Inconsistent evidence	Low–moderate

**Table 2:** Pharmacological strategies for acute treatment of perioperative arrhythmias.

Drug/Class	Mechanism	Indication	Clinical Use	Benefits	Risks/Limitations	Level of Evidence
Beta-blockers (esmolol, metoprolol)	↓ AV conduction, ↓ sympathetic tone	AF/flutter rate control; SVT	First-line in stable patients	Effective, titratable (short-acting agents)	Hypotension, bradycardia	High (guidelines, RCTs)
Calcium channel blockers (diltiazem, verapamil)	AV nodal blockade	AF/flutter rate control	Alternative to β-blockers	Effective ventricular rate control	Negative inotropy, hypotension	High (guidelines)
Amiodarone (therapeutic)	Multichannel blockade	AF (rate/rhythm), VT	When β-blockers/CCB fail or LV dysfunction present	Broad efficacy; safe in LV dysfunction	Hypotension, QT prolongation	High
Digoxin	↑ vagal tone, AV node inhibition	AF rate control (adjunct)	Second-line or in HF patients	Minimal hypotension	Slow onset; less effective in stress states	Moderate
Adenosine	Transient AV block	Regular narrow-complex SVT	Diagnostic and therapeutic bolus	Rapid termination of SVT	Bronchospasm, transient asystole	High
Procainamide	Class Ia; slows conduction	Stable wide-complex tachycardia	Selected monitored settings	Effective in VT and pre-excited arrhythmias	Hypotension, QT prolongation	Moderate
Lidocaine	Class Ib; Na^+^ channel blockade	Ventricular arrhythmias (ischaemia-related)	Alternative to amiodarone in VT	Useful in ischaemic substrate	Limited scope	Moderate
Magnesium sulfate	Stabilises repolarization	Torsade de pointes; QT prolongation	First-line in TdP	Highly effective, safe	Limited outside TdP	High
Atropine	Anticholinergic	Symptomatic bradycardia	First-line bradycardia	Rapid action	Ineffective in infranodal block	High
Vasopressors/inotropes	Adrenergic stimulation	Bradycardia with shock	Bridge therapy	Maintains perfusion	May trigger arrhythmias	Low–moderate
Anticoagulation (DOACs, warfarin)	Thrombin/Xa inhibition	Stroke prevention in POAF	Based on CHA_2_DS_2_-VASc and bleeding risk	Reduces thromboembolism	Bleeding risk; timing challenges	High (guidelines, RCTs)
Lipid emulsion	“Lipid sink”	Local anaesthetic toxicity arrhythmias	Emergency LAST management	Life-saving	Not routine use	High (guidelines, consensus)

**Table 3:** Representative non-antiarrhythmic QT-prolonging drugs commonly encountered in the perioperative and critical-care setting, with torsade de pointes (TdP) risk and practical mitigation notes.

Drug Class (Examples)	Typical Perioperative Use	TdP Risk (CredibleMeds)	Practical Considerations
Macrolides (azithromycin, clarithromycin, erythromycin)	Infection treatment/prophylaxis	Known TdP risk	Prefer non-macrolide alternatives where feasible; clarithromycin and erythromycin also inhibit CYP3A4, amplifying co-administered QT-active drugs
Fluoroquinolones (moxifloxacin, levofloxacin, ciprofloxacin)	Infection treatment	Known/possible risk (moxifloxacin highest; ciprofloxacin lowest)	Favour ciprofloxacin when a fluoroquinolone is needed in high-risk patients; avoid moxifloxacin
Azole antifungals (fluconazole, voriconazole)	Invasive fungal infection	Known/possible risk	Dose-dependent effect; potent CYP inhibitors that potentiate other QT-active drugs
Antipsychotics/butyrophenones (haloperidol, droperidol)	Delirium, agitation, antiemesis	Known/conditional risk (greatest with high-dose intravenous use)	ECG before and after intravenous use; risk highest with the IV route and cumulative dosing
Antiemetics (ondansetron, droperidol)	Postoperative nausea and vomiting prophylaxis	Known/conditional risk	Dose-related (single IV ondansetron dose ≤ 16 mg); avoid stacking with other QT-active drugs
Antidepressants (citalopram, escitalopram, tricyclics)	Chronic therapy continued perioperatively	Known/possible risk	Observe citalopram/escitalopram dose ceilings; consider baseline ECG, particularly with polypharmacy
Methadone	Chronic opioid therapy/opioid-use disorder	Known TdP risk	Dose-dependent; baseline and follow-up ECG; caution when combined with other QT-active drugs
First-generation antihistamines	Sedation, antiemesis, premedication	Conditional/possible risk	Use cautiously in long QT syndrome and with concurrent QT-prolonging agents

TdP risk categories follow the AZCERT CredibleMeds QTdrugs classification (known, possible, or conditional risk of TdP) and are periodically updated [45]. CYP3A4, cytochrome P450 3A4; ECG, electrocardiogram; IV, intravenous; TdP, torsade de pointes.

## Data Availability

No new data were generated or analysed in support of this research.

## Abbreviations

ACC, American College of Cardiology; ACCP, American College of Clinical Pharmacy; ACLS, advanced cardiac life support; AF, atrial fibrillation; AHA, American Heart Association; AI, artificial intelligence; AUC, area under the curve; AV, atrioventricular; BNP, B-type natriuretic peptide; CABG, coronary artery bypass grafting; CI, confidence interval; CIED, cardiac implantable electronic device; CO, cardiac output; COPD, chronic obstructive pulmonary disease; CPVT, catecholaminergic polymorphic ventricular tachycardia;CYP3A4, cytochrome P450 3A4; DOAC, direct oral anticoagulant; EACTA, European Association of Cardiothoracic Anaesthetists; ECG, electrocardiogram; EHR, electronic health record; EMI, electromagnetic interference; ESAIC, European Society of Anaesthesiology and Intensive Care; ESC, European Society of Cardiology; HERG, human ether-related gene; HF, heart failure; HR, hazard ratio; hs-CRP, high-sensitivity C-reactive protein; HRS, Heart Rhythm Society; ICa-L, L-type calcium current; ICD, implantable cardioverter-defibrillator; IKACh, acetylcholine-activated potassium current; IKr, rapid delayed-rectifier potassium current; IKs, slow delayed-rectifier potassium current; IL-1, interleukin-1; IL-6, interleukin-6; ILR, implantable loop recorder; INa, cardiac sodium current; Ito, transient outward potassium current; LAST, local anaesthetic systemic toxicity; LQTS, long QT syndrome; LV, left ventricular; MAC, minimum alveolar concentration; ML, machine-learning; NSQIP, National Surgical Quality Improvement Program; NSVT, non-sustained ventricular tachycardia; NT-proBNP, N-terminal pro–B-type natriuretic peptide; OR, odds ratio; POAF, postoperative atrial fibrillation; PPG, photoplethysmography; PVC, premature ventricular complex; QRS, QRS complex; QTc, corrected QT interval; QTd, QT dispersion; RCRI, Revised Cardiac Risk Index; RCT, randomised controlled trial; RR, risk ratio; SCA, Society of Cardiovascular Anesthesiologists; SQTS, short QT syndrome; SVT, supraventricular tachycardia; TdP, torsade de pointes; TEE, transoesophageal echocardiography; VF, ventricular fibrillation; VT, ventricular tachycardia.
